# Effects of Dielectric Interlayer on Polarization Switching and Rectifying Characteristics in Al_0.8_Sc_0.2_N/HfO_2_ Ferroelectric Diodes

**DOI:** 10.3390/mi17060742

**Published:** 2026-06-19

**Authors:** Jong Min Park, Hyeong Jun Joo, Yoojin Lim, Juno Bae, Brendan Hanrahan, Geonwook Yoo

**Affiliations:** 1 Department of Intelligent Semiconductor, Soongsil University, Seoul 06938, Republic of Korea; uw5688@soongsil.ac.kr (J.M.P.); bugsholic@soongsil.ac.kr (H.J.J.); lyjddw@soongsil.ac.kr (Y.L.); bjo0608@soongsil.ac.kr (J.B.); 2 DEVCOM Army Research Laboratory, Adelphi, MD 20783, USA; brendan.m.hanrahan.civ@army.mil

**Keywords:** AlScN, ferroelectric diode, metal–insulator–ferroelectric-metal, interfacial, oxygen substitutional defect

## Abstract

Ferroelectric (FE) diodes configured in the metal–ferroelectric–metal (MIFM) structure are promising candidates for non-volatile memory. While recent studies emphasized bulk FE properties, interfacial characteristics have not been carefully considered. In this work, we investigate the HfO_2_/Al_0.8_Sc_0.2_N interface by examining its impact on switching and rectifying characteristics in MIFM FE diodes with variable HfO_2_ thicknesses (2/4/6 nm). Electrical characterization reveal that the increased HfO_2_ thickness raises the coercive field (E_C_) due to enhanced electrostatic effects and progressive interfacial oxidation from Sc-N to Sc-O bonds. This resulting oxygen substitutional defect (O_N_) which may contribute to domain-wall pinning and reduced rectifying efficiency. Cycling tests clarify operating regime-dependent phenomena, including O_N_ redistribution-induced wake-up and eventual breakdown. Moreover, enhanced retention is observed after pre-cycling, originating from the stabilization of the interfacial defects rather than bulk properties. These findings underscore that E_C_ and device reliability are likely influenced by interfacial engineering, which is critical for the reliable operation of AlScN-based FE diodes.

## 1. Introduction

As the demand for low-power, scalable, and non-volatile memory devices intensifies, ferroelectric (FE) materials including fluorites and recent wurtzites, such as aluminum scandium nitride (Al_1−x_Sc_x_N), have emerged as key enablers due to their robust polarization characteristics and integration compatibility with CMOS platforms [1,2]. These materials offer tunable remnant polarization (P_r_), suppressed leakage currents, and high endurance, making them suitable for next generation. FE-based non-volatile memory (NVM) technology capable of multi-state operations, thereby enabling advanced analog computing and neuromorphic computing applications [3,4]. Among these, AlScN stands out for its large P_r_ value (~100 μC/cm^2^) and coercive field (E_C_) typically in the range of 5–6 MV/cm, as well as a wide bandgap ranging from 4.3 to 6.12 eV depending on the Sc content [5,6]. Alongside AlScN, HfO_2_-based FE materials have been widely explored for FE diode applications [7] as well as versatile applications such as memristive artificial synapses and neuromorphic computing systems [8,9]. However, HfO_2_-based FE diodes are constrained by thermal limitations, as they typically require pre-conditioning steps, such as high temperature annealing, and prolonged exposure to elevated temperatures can induce phase transitions that compromise ferroelectric phase stability. In contrast, AlScN inherently exhibits as-grown ferroelectricity, which originates from the wurtzite lattice distortion and enables stable, wake-up-free switching [10]. Additionally, its high curie temperature (~1150 °C) provides excellent thermal stability, making it attractive for harsh environment applications [11]. In addition to these intrinsic properties, AlScN offers excellent compatibility with CMOS BEOL processes, which serves as a significant advantage particularly for integration in RF filters and FE microelectronic applications [12]. In addition, AlScN has recently been applied to three-terminal devices such as FE HEMTs, demonstrating its potential for logic–memory integration and reliable operation in harsh environments [13,14].

Among various FE-based NVM devices, FE diode has been investigated as its self-selecting structure for high-density memory [15,16,17,18]. Polarization-controlled modulation of Schottky barrier at the metal/FE interface enables diode-like characteristics. A metal/dielectric interlayer/ferroelectric/metal (MIFM) structure has been widely investigated for the FE diodes. This configuration facilitates modulation of an internal electric field and electrode interface, making it highly suited for investigating polarization switching, interface charge modulation, and carrier transport behavior [19,20]. For AlScN-based MIFM stacks, in particular, spontaneous polarization and large coercive field (E_C_) generate a strong internal field that can naturally lead to a current–voltage (I–V) asymmetry [21]. Additionally, inserting a dielectric (DE) interlayer between an electrode and FE layer has been explored to enhance endurance and thermal stability of FE switching [22]. However, such modifications may also introduce asymmetries in carrier injection, depolarization fields (E_dep_), and thus switching characteristics [23]. Ultimately, this study elucidates how interfacial and defect-mediated phenomena, beyond bulk properties, govern the complex electrical behavior and rectifying characteristics of HfO_2_/AlScN FE devices. It further highlights the critical role of stacking configuration in determining FE crystallinity and the resulting device performance. This fundamental understanding, revealing the origins of variations in FE properties and their direct manifestation as rectification, provides crucial insights for the rational design and optimization of future high-performance, high-reliability FE devices.

In this work, we investigate the structure of AlScN-based MIFM FE diodes. Crystallinity was examined by X-ray diffraction (XRD), while polarization hysteresis and charge-modulation behavior revealed stable FE switching. Rectifying characteristics of the current were analyzed, showing asymmetry correlated with the strength of polarization switching. Electrical cycling was carried out to observe wake-up behavior, and retention characteristics before and after pre-cycling were compared to examine the influence of cycling on polarization stability.

## 2. Materials and Methods

Al_0.8_Sc_0.2_N-based MIFM and MFIM diodes were fabricated. For the MIFM devices, the diode stack consisted of a Ti/Pt (5 nm/50 nm) bottom electrode (BE), an AlScN (70 nm) FE layer, an HfO_2_ (2, 4, and 6 nm) dielectric (DE) interlayer, and a Ni (50 nm) top electrode (TE). For the MFIM device, the stack consisted of a Ti/Pt (5 nm/50 nm) BE, an HfO_2_ (6 nm) DE interlayer, an AlScN (70 nm) FE layer, and a Ni (50 nm) TE.

The Ti/Pt BE was deposited on a heavily p-doped Si substrate by reactive sputtering at room temperature for both MIFM and MFIM devices. The AlScN films were deposited for both device structures simultaneously by reactive RF magnetron sputtering using a single Al–Sc alloy target. Deposition was performed at a set temperature of 400 °C in an Ar/N_2_ ambient with flow rates of 15 and 30 sccm, respectively, under a chamber pressure of 5 mTorr. The HfO_2_ films with a thickness of 2, 4, and 6 nm were deposited at 350 °C by thermal-ALD using tetrakis-(ethylmethylamido)hafnium as a precursor and ozone (O_3_) as a reactant. The deposition rate was 1.63 Å/Cycle, corresponding to 12, 24, and 36 cycles for 2, 4, and 6 nm, respectively. Ni (50 nm) TE with a diameter of 30 µm was deposited and patterned simultaneously for both MIFM and MFIM devices by a deposition and lift-off process.

For structural and chemical analyses, XRD (Bruker, Billerica, MA, USA, D8 Advance) and X-ray pho-toelectron spectroscopy (XPS) (Thermo Fisher, Waltham, MA, USA, Scientific K-Alpha+) with Ar ion sputtering were employed. Electrical measurements were conducted to evaluate FE and DE properties. Polarization–voltage (P–V) loops and switching-current transients were measured using a pulse-measurement unit (Keithley, Solon, OH, USA, PMU-4225) and a FE tester (Radiant, Albuquerque, NM, USA, RT66C). Capacitance–voltage (C–V) characteristics were measured using a precision LCR meter (Hewlett-Packard, Palo Alto, CA, USA, 4284A). Steady-state current-density–voltage (J–V) characteristics under DC bias were obtained using a semiconductor characterization system (Keithley, Solon, OH, USA, 4200A-SCS).

## 3. Results

**Figure 1:**
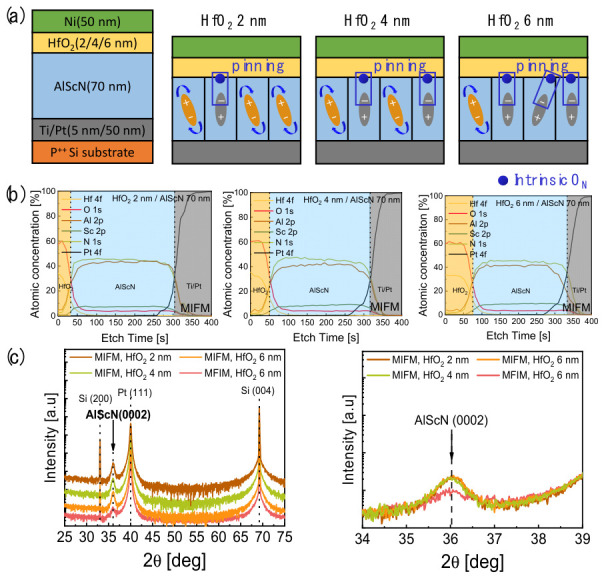
(**a**) Schematic illustration of O_N_-mediated domain-wall pinning at the HfO_2_/AlScN interface. A thicker HfO_2_ interlayer has increased pinning centers and suppressed switching. The blue curved arrows indicate switchable ferroelectric dipoles. (**b**) XPS depth profiles of the Ni/HfO_2_/AlScN/Ti/Pt stack with a dielectric interlayer thickness of 2, 4, and 6 nm. (**c**) XRD θ–2θ scans with the AlScN (0002) reflection at 36°.

Figure 1 presents the fabricated MIFM structure with structural analysis results of XPS and XRD. Figure 1a schematically illustrates that increasing the HfO_2_ interlayer thickness leads to a higher density of oxygen substitutional defect (O_N_)-mediated pinning centers at the HfO_2_/AlScN interface. Generally, during oxidation of AlN, nitrogen lattice sites are vacated and subsequently occupied by oxygen atoms [24]. In AlScN, the incorporation of Sc modifies this oxidation pathway. Because replacing a Sc–N bond with a Sc–O bond yields a larger energy gain than replacing an Al–N bond with an Al–O bond, oxygen preferentially substitutes nitrogen sites adjacent to Sc ions under oxidative conditions [25]. Nevertheless, oxidation still proceeds through substitution of nitrogen sites by oxygen, resulting in the formation of O_N_s within the AlScN lattice. These defects introduce excess electrons and therefore behave as negatively charged centers. In the as-deposited state, such localized charged defects can act as pinning sites that hinder domain nucleation and wall motion, thereby suppressing FE switching [26]. Figure 1b shows depth-profiled XPS spectra confirming the Ni/HfO_2_/AlScN/Ti/Pt stack; An Al:Sc composition ratio of about 80:20 was obtained from the etch depth corresponding to the mid-position of the AlScN layer, where the composition remained nearly constant. Figure 1c presents XRD θ–2θ scans, showing that the (0002) diffraction peak near 36° is preserved. For the MIFM structures, the (0002) diffraction peak is consistently observed near 36° for all samples with comparable peak characteristics. This indicates that the intrinsic crystalline quality and bulk properties of the AlScN film are essentially unchanged across the MIFM devices. In contrast, the AlScN (0002) peak intensity in the MFIM structure is relatively weaker than that in the MIFM structures, likely because the amorphous ALD-grown HfO_2_ layer serves as a non-crystalline growth surface that limits the crystalline quality of the subsequently deposited AlScN film, suggesting degraded FE characteristics. Based on these structural and chemical observations, we next evaluate the switching dynamics using positive-up– negative-down (PUND) measurements.

**Figure 2:**
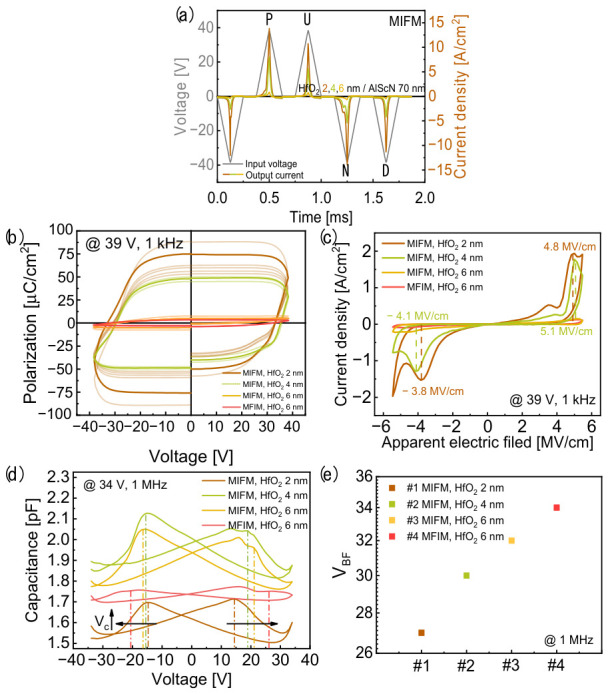
(**a**) Pulse scheme of the positive-up–negative-down (PUND) measurement with representative measured output currents at 39 V. (**b**) P–V loops measured by PUND, and (**c**) J–E characteristics at 39 V and 1 kHz. (**d**) C–V curves measured at 34 V and 1 MHz. The vertical dashed lines indicate the coercive voltage points corresponding to each sample. (**e**) Onset voltage of the C–V characteristics for devices with different DE thicknesses.

Figure 2a–c present the switching characteristics of MIFM devices with HfO_2_ DE interlayer thicknesses of 2, 4, and 6 nm, measured by PUND at 39 V and 1 kHz. As the HfO_2_ thickness increased from 2 to 4 nm, the apparent E_C_ increased from 4.30 to 4.60 MV/cm. The apparent E_C_ is defined as the average field position of the positive- and negative-polarity switching-current peaks in the J–E response. It should be noted that the reported E_C_ is obtained by dividing the applied voltage by the total stack thickness, since the CIS model cannot be readily applied to extract V_FE_ via voltage division in the present DE/FE bilayers, as discussed below. It should be noted that the apparent E_C_ reported here reflects the device-level switching field of the entire stack rather than the intrinsic E_C_ of the AlScN layer alone. Notably, two switching-current peaks appear in the positive field region in Figure 2c, attributable to non-uniform internal fields arising from charged defects or trapped charges within the FE layer or at the DE/FE interfaces [27,28]; E_C_ is defined using the higher-field peak, as it corresponds to the dominant switching. Both switching current and P_r_ decreased with increasing DE thickness, and no switching was observed at these pulse conditions for the 6 nm HfO_2_ device in either the MIFM or a simultaneously fabricated MFIM structure. The observed increase in apparent E_C_ with HfO_2_ thickness is considerably smaller than that expected from the CIS model, in which sufficient interfacial charge compensation decouples the FE and DE layers and produces a well-defined voltage division [29]. This limited thickness dependence of E_C_ suggests that such effective CIS operation is not realized in the present DE/FE bilayers, likely due to insufficient interfacial charge accumulation. Figure 2d shows butterfly shaped C–V curves measured at 34 V and 1 MHz. The butterfly peak voltage shifts to higher values with increasing DE thickness, and the MFIM structure exhibits a larger apparent coercive voltage (V_C_) than the MIFM devices. Additionally, two peaks are also observed in the positive voltage region of the C–V curves, which is likely attributable to the same physical origin as discussed in the J–E characteristics in Figure 2c. In contrast, these two peaks are not observed in the MFM control device, consistent with the absence of DE/FE interfacial defects that are likely responsible for the non-uniform internal fields in the MIFM and MFIM structures. As shown in Figure 1c, the weaker AlScN (0002) peak intensity in the MFIM structure indicates reduced crystalline quality, which likely weakens FE switching and raises V_C_. As shown in Figure 2e, the onset of the butterfly C–V curves, denoted as V_BF_ occurs at 27, 30, and 32 V for DE thicknesses of 2, 4, and 6 nm, respectively, indicating that a thicker DE layer requires a higher effective switching voltage. Meanwhile, the zero-bias capacitance shows a non-monotonic dependence on DE thickness, peaking at 4 nm, whereas a simple CIS model predicts a monotonic decrease, consistent with the corresponding equivalent oxide thickness values calculated for the HfO_2_/AlScN (70 nm) stacks: 14.4 nm (2 nm), 15.8 nm (4 nm), and 16.2 nm (6 nm). This deviation from the CIS prediction can be attributed to electrostatic coupling in FE/DE bilayers. When the HfO_2_ thickness is 2 nm, the E_dep_ is relatively weak, and the FE layer is expected to maintain stable polarization with relatively lower sensitivity to external fields, resulting in lower capacitance. As the HfO_2_ thickness increases to 4 nm, the strengthened E_dep_ becomes comparable to the FE polarization, placing the system in a state where polarization responds most sensitively to external fields. This leads to a maximum in dP/dE and the observed capacitance peak [30]. Beyond this point, the further increasing E_dep_ with thicker HfO_2_ is expected to progressively suppresses polarization susceptibility, resulting in reduced capacitance for the 6 nm device. This non-monotonic capacitance behavior, together with the limited thickness dependence of apparent E_C_, suggests that the bilayer does not behave as a simple series-capacitor stack but rather as an electrostatically coupled system.

**Figure 3:**
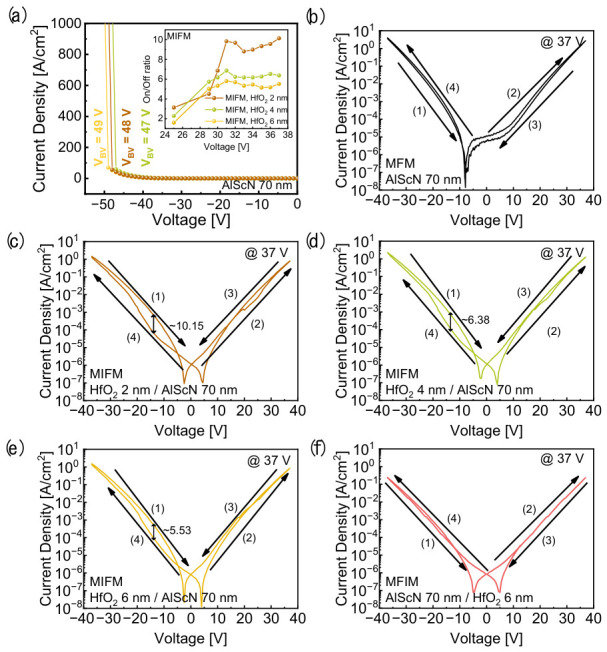
(**a**) Breakdown voltage characteristics of MIFM devices with HfO_2_ thicknesses of 2, 4, and 6 nm. Inset: Extracted on–off ratio as a function of applied voltage (25–37 V). The numbers (1)–(4) indicate the sequence of the voltage sweep. (**b**–**f**) J–V characteristics measured at 37 V.

Rectifying J–V properties of FE diode are presented in Figure 3. Current rectification originates from asymmetric top and bottom electrodes, whose work function difference enables polarization reversal to modulate the relative Schottky barriers [7,31,32]. Inserting a DE interlayer redistributes the internal electrostatic potential and amplifies the barrier modulation, thereby exhibiting diode-like responses. The breakdown voltage showed no significant dependence on HfO_2_ thickness, measuring approximately 47–49 V across all devices as shown in Figure 3a, indicating that dielectric breakdown is likely governed by the AlScN bulk layer rather than the ultrathin HfO_2_ interlayer. As shown in the inset of Figure 3a, the On/Off ratio saturates above ~31 V; thus, the subsequent J–V data presented in Figure 3b–f were obtained at 37 V to ensure fully switched FE conditions. As shown in Figure 3b, the MFM control device does not exhibit discernible rectifying behavior and shows comparatively higher leakage current than the MIFM and MFIM devices, further corroborating the critical role of the DE interlayer in enabling rectification. Although a thicker DE interlayer is generally expected to suppress leakage current, the measured current density remains nearly unchanged for 2, 4, and 6 nm HfO_2_. This behavior can be understood as a balance between thickness-driven suppression of leakage pathways and nitrogen vacancy (V_N_)-induced conduction band bending at the HfO_2_/AlScN interface, which lowers the effective Schottky barrier and promotes defect-assisted transport [33]. Under fully switched conditions, degradation of rectification with the thicker DE interlayer was observed, likely related to weakened polarization-induced barrier modulation. The On/Off ratio decreased from 10.15 (2 nm device) to 6.38 (4 nm device) and further to 5.53 (6 nm device) in the negative bias region, accompanied by an increasing overlap between the forward and reverse sweeps under positive bias. These findings are consistent with the results of switching characteristics, and the correlation between reduced polarization strength and rectification degradation can be further elaborated through energy-band diagram analysis.

**Figure 4:**
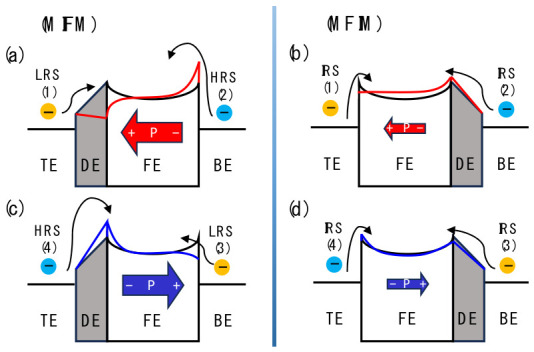
Schematic energy-band diagrams of (**a**,**c**) MIFM structure and (**b**,**d**) a comparative MFIM structure under different polarization states. (**a**,**b**) represent upward polarization, and (**c**,**d**) correspond to downward polarization. Compared with MIFM, the MFIM structures exhibit weaker switching and reduced band bending.

Figure 4 illustrates the operating principle of FE diodes using schematic energy-band diagrams of the MIFM and MFIM structures. The difference in rectification between the MIFM and MFIM devices is primarily driven by the switched polarization, which asymmetrically modulates the Schottky barrier heights. In Figure 4a, upward polarization under negative bias at the top electrode lowers the interfacial barrier and yields a low-resistance state (LRS, state 1), whereas reversing bias increases the barrier and thus produces a high-resistance state (HRS, state 2). Conversely, in Figure 4c, downward polarization favors LRS (state 3) under positive bias and HRS (state 4) under negative bias. These four states correspond to the numbered conditions (1)–(4) in Figure 3b–e. Within the MIFM devices, a thicker DE interlayer requires a higher apparent switching voltage, so the switchable polarization and, consequently, the barrier modulation decreases. In contrast, the MFIM structure in Figure 3f does not exhibit a distinct transition between rectifying states, suggesting that the threshold of polarization reversal exceeds the applicable voltage range. The device therefore remains in an intermediate-resistance state (IRS) with nearly symmetric conduction. In the conventional MFM structure, the polarization-bound charges at the FE surface are effectively screened by the metal electrodes. In the MIFM structure, however, the insertion of a DE interlayer may leave these bound charges only partially compensated, which could give rise to a E_dep_ within the FE layer. To accommodate this E_dep_, the FE layer is likely to develop a multi-domain configuration, which is expected to become denser as the DE thickness increases. Such a denser domain configuration may reduce the effective driving field experienced by the FE layer at a given applied bias, so that a larger external voltage could be required to trigger polarization reversal [34]. Moreover, interfacial defects, including O_N_ at the HfO_2_/AlScN interface may induce domain-wall pinning. This speculation is further supported by subsequent electrical measurements. Regarding the origin of this interfacial oxidation, the deposition of AlScN and HfO_2_ was not performed in situ, and thus the formation of a native oxide at the AlScN surface cannot be excluded. However, all MIFM samples experienced identical air exposure before the ALD process, indicating that native oxide contribution is comparable across all devices. Meanwhile, during the ALD deposition of HfO_2_, interfacial oxidation induced by high-temperature O_3_ exposure increases as the DE interlayer thickness increases.

Accordingly, the observed increase in apparent E_C_ can be reasonably interpreted as thickness-dependent interfacial effects arising from the ALD process conditions, rather than from an uncontrolled native oxide layer.

**Figure 5:**
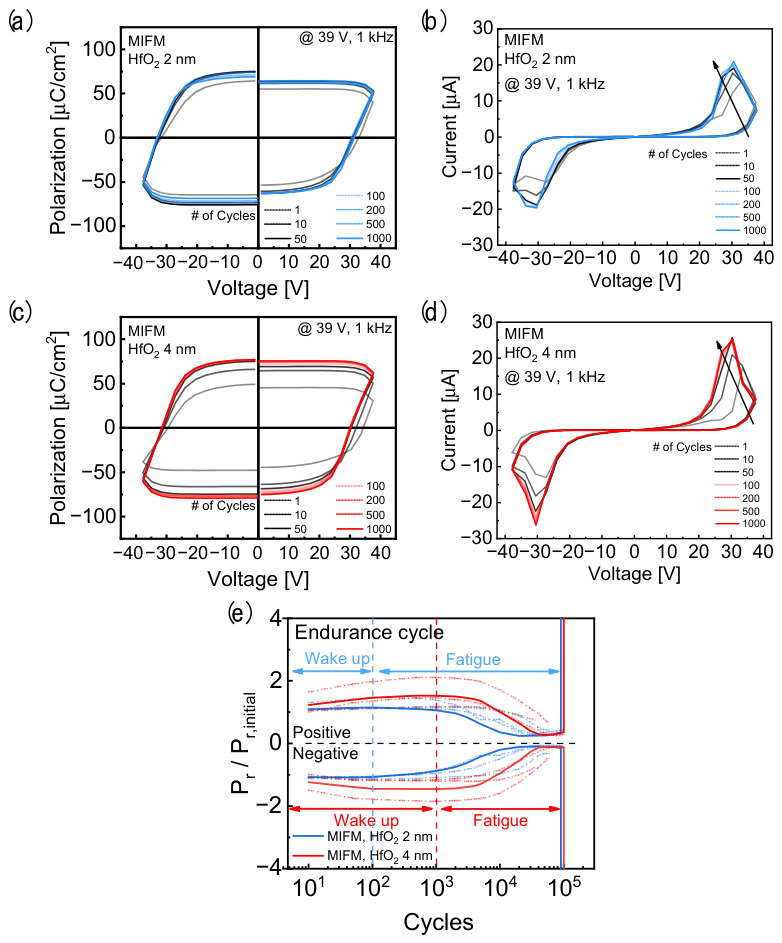
Endurance characteristics of MIFM FE diodes measured under a bipolar triangular waveform (±39 V, 1 kHz). P-V loops and switching-current characteristics as a function of cycling for the (**a**,**b**) 2 nm and (**c**,**d**) 4 nm devices. The arrow indicates the switching current peak shift over cycles; colors match the legend. (**e**) Comparison of normalized polarization versus cycle number.

Figure 5 shows endurance characteristics of the FE diodes with 2 nm and 4 nm HfO_2_, evaluated through cycling under a bipolar triangular waveform (± 39 V, 1 kHz). It is to be noted that only 2 nm and 4 nm devices were examined because the thicker DE interlayer (6 nm) requires a high switching voltage beyond the 39 V limit of the PMU-4225 measurement unit. The 2 nm and 4 nm devices, however, represented distinct switching and defect-activity regimes, showing fully switched and partially switched states within the measurable voltage range. These differences allowed meaningful analysis of field-cycling-induced behavior. As shown in Figure 5a,b, the 2 nm device maintained stable polarization and current characteristics during cycling. The polarization slightly increased from 65 to 75 μC/cm^2^. In contrast, the 4 nm device exhibited larger variations in both current and polarization, which increased from 48 to 76 μC/cm^2^, as presented in Figure 5c,d. This pronounced change in polarization is indicative of wake-up behavior, as further confirmed in Figure 5e, which summarizes the polarization change with cycling. Despite these differences, both FE diodes maintained endurance up to approximately 10^5^ cycles with comparable breakdown strength. The observed wake-up in the 4 nm device can be associated with two factors. First, cycling under a field not exceeding the apparent E_C_ progressively activates unswitched domains, which is characteristic of minor-loop operation [35]. Second, defect-mediated processes may also contribute. Exposure to O_3_ reactants during HfO_2_ deposition can modify the AlScN interface and promote formation of O_N_.

**Figure 6:**
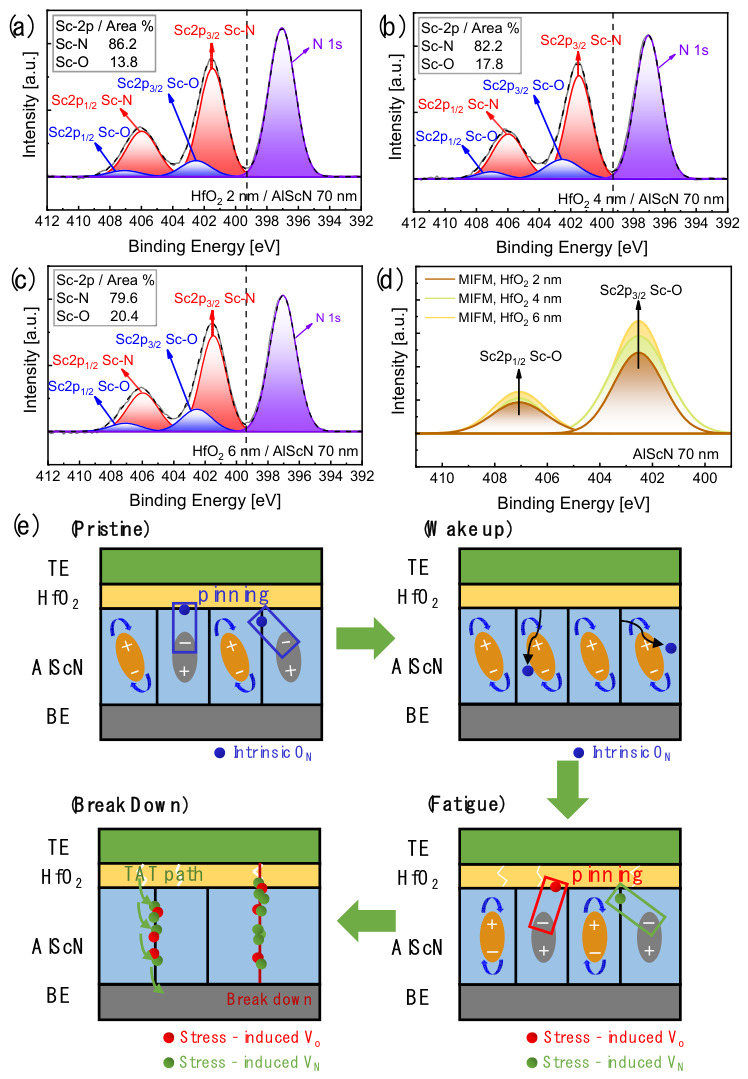
XPS analysis of the HfO_2_/AlScN interface showing the relative fractions of Sc–N and Sc–O bonds as a function of binding energy for MIFM FE diodes with HfO_2_ interlayers of (**a**) 2 nm, (**b**) 4 nm, and (**c**) 6 nm. (**d**) Comparison of the Sc–O bonding fractions extracted from (**a**–**c**), (**e**) Schematic illustration of the proposed mechanism for cycling-induced behavior during wake-up, fatigue, and breakdown. The solid lines represent domain walls, and the thick green arrows indicate the sequential progression of phenomena during cycling.

In hafnia-based FE, oxygen vacancy (V_O_) has been reported to act as domain-wall pinning centers, increasing apparent E_C_ and reducing reversibility. The redistribution and partial regeneration of such defects under repeated cycling are widely regarded as the primary origin of wake-up behavior [28,36]. By analogy, O_N_ in the AlScN are likely to behave in a similar manner. While O_N_ in AlScN is a negatively charged substitutional defect and differs from V_O_ in its charge state and migration dynamics, it may play a phenomenologically similar role in domain-wall pinning. Specifically, domain-wall motion is expected to locally reduce the migration barrier of O_N_ defects, enabling short-range hopping under electric fields exceeding approximately 1.5 MV/cm [26]. Since our devices operate well above this threshold, field-driven local redistribution of ONs is expected to provide a physically plausible mechanism for the observed wake-up and fatigue behavior. The detailed dynamics of how the redistribution of these substitutional defects progressively enables de-pinning are illustrated in Figure 6e.

In this context, XPS analysis was conducted to characterize compositional changes at the interface. Figure 6a–c present the XPS core-level spectra measured near the HfO_2_/AlScN interface for each structure. For XPS peak fitting, the interfacial region was identified at the etch depth where the Hf-4f and Sc-2p signals exhibited comparable intensities. The Sc-2p and N-1s core levels were fitted using standard reference peak positions for Sc–N and Sc–O bonds [37,38]. As the number of ALD cycles increased—corresponding to longer O_3_ exposure during HfO_2_ deposition—the fraction of Sc-N bonds decreased, while the Sc–O fraction rose from 13.8% (2 nm HfO_2_) to 17.8% (4 nm HfO_2_) and 20.4% (6 nm HfO_2_) as summarized in Figure 6d. The observed decrease in Sc-N bonds accompanied by an increase in Sc-O bonds suggests generation of O_N_ within the AlScN layer near the interface. Such spectroscopic evidence is consistent with the thickness-dependent increase in extracted E_C_. The schematic in Figure 6e summarizes the defect dynamics during cycling. In the pristine state, pre-existing O_N_ defects at or near the HfO_2_/AlScN interface are likely to act as charged pinning centers, creating a local internal bias field that hinders domain-wall motion. The subsequent wake-up stage is attributed to two possible factors: operation below E_C_ and the redistribution of charged defects under field cycling. In particular, the field-assisted migration of O_N_ defects toward energetically more stable configurations can partially relax the internal bias field and reduce domain pinning, thereby facilitating polarization switching. During the fatigue stage, although oxygen diffusion along grain boundaries has been identified as a primary degradation mechanism in AlScN-based ferroelectrics exposed to ambient conditions [39], the top HfO_2_ layer in the present MIFM structure is expected to serve as a passivation barrier, making this pathway less dominant. Instead, repeated field cycling can generate or activate V_N_ within the AlScN layer and V_O_ within the degrading HfO_2_ interlayer, reinforcing domain pinning and increasing leakage current [40]. Associated Joule heating further accelerates vacancy generation and redistribution in both layers, thereby diminishing switchable polarization. Finally, breakdown occurs when vacancy-related defects in the AlScN and HfO_2_ layers percolate along grain boundaries, forming continuous trap-assisted tunneling paths between the electrodes.

**Figure 7:**
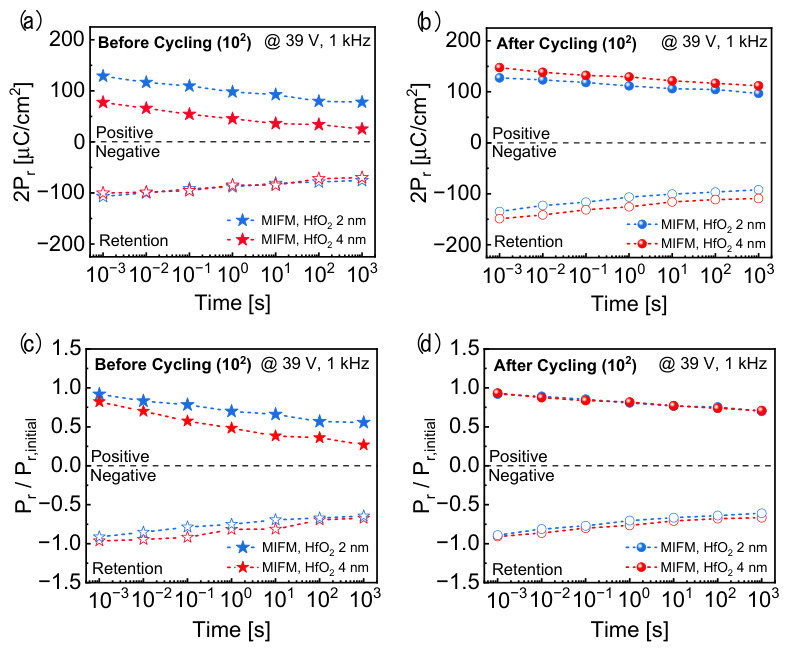
Retention characteristics of MIFM FE diodes with 2 and 4 nm HfO_2_ interlayers measured up to 10^3^ s (39 V, 1 kHz). (**a**,**b**) 2P_r_ values before and after 100 cycles, respectively. (**c**,**d**) Normalized polarization (P_r_/P_r,initial_) over time before and after 100 cycles. The star and circle symbols indicate the data before and after 100 cycles, respectively.

Retention characteristics of the MIFM FE diodes are characterized up to 10^3^ s. Figure 7a,c present the results prior to 100 cycles. Both 2 nm and 4 nm devices exhibited degradation, which is more pronounced for the 4 nm device. Retention degradation in the MIFM FE diodes is generally ascribed to the enhanced E_dep_ arising from incomplete screening of surface-bound charges across the inserted DE layer [41]. After performing pre-cycling (100 cycles), however, the retention characteristics exhibit a remarkable improvement. Notably, the observed discrepancy becomes less distinct after pre-cycling, consistent with the polarization evolution observed in Figure 5. As discussed for Figure 5 and Figure 6, two possible mechanisms explain the improvement of retention, and validate consistency between the two different measurements.

Finally, comparison between pre- and post-cycling retention in the MIFM devices suggests that the intrinsic polarization of the 70 nm AlScN layer remains unchanged, consistent with the absence of any shift in the (0002) XRD peak in Figure 1. Therefore, the discrepancy in retention characteristics is reasonably attributed to variations in the apparent E_C_ induced by the combined influence of the E_dep_, and interfacial defects. In this context, the 4 nm FE diode can be regarded as having a higher E_C_ than the 2 nm device under the same operating condition. Therefore, these retention results, in addition to the observed apparent E_C_ difference, underscore the importance of interfacial defect control as a prerequisite for ensuring reliable device operation.

## 4. Conclusions

In conclusion, we have investigated the origins of the observed switching characteristics by comprehensively analyzing wake-up, fatigue, and breakdown across successive cycling, together with retention behavior under pre- and post-cycling conditions. Structural analysis supports interfacial oxidation and substitutional oxygen defect-related processes as key contributing factors beyond simple voltage-division or E_dep_. While prior studies have largely emphasized high-performance metrics of FE diodes, the results elucidate why such behaviors emerge and highlight the potential roles of O_N_, interfacial chemistry, and defect redistribution. Furthermore, the observed improved retention characteristics after pre-cycling suggest that controlled electrical stressing can be used as a practical method to redistribute O_N_ and stabilize the interfacial pinning for long-term reliability. These insights point to the importance of controlling interface conditions for the AlScN-based FE diode, suggesting that key characteristics, such as the apparent coercive field and reliability metrics, are likely dependent on interfacial engineering rather than bulk properties alone. In addition to the superior properties of AlScN, our findings underscore exploring defect–ferroelectric interactions for interface engineering strategies in wurtzite ferroelectrics.

## Data Availability

The original contributions presented in this study are included in the article. Further inquiries can be directed to the corresponding author.
